# The relationship between cardiopulmonary exercise test variables, the systemic inflammatory response, and complications following surgery for colorectal cancer

**DOI:** 10.1186/s13741-018-0093-8

**Published:** 2018-06-15

**Authors:** Stephen T. McSorley, Campbell S. D. Roxburgh, Paul G. Horgan, Donald C. McMillan

**Affiliations:** Academic Unit of Surgery, School of Medicine, University of Glasgow, R2.06, Level 2, New Lister Building, Glasgow Royal Infirmary, Alexandra Parade, Glasgow, G31 2ER UK

**Keywords:** Colorectal cancer, Cardiopulmonary exercise testing, Systemic inflammation

## Abstract

**Background:**

Both preoperative cardiopulmonary exercise test (CPET)-derived measures of fitness and postoperative C-reactive protein (CRP) concentrations are associated with complications following surgery for colorectal cancer. The aim of the present pilot study was to examine the relationship between CPET and postoperative CRP concentrations in this patient group.

**Methods:**

Patients who had undergone CPET prior to elective surgery for histologically confirmed colorectal cancer in a single centre between September 2008 and April 2017 were included. Preoperative VO_2_ at the anaerobic threshold (AT) and peak exercise were recorded, along with preoperative modified Glasgow Prognostic Score (mGPS) and CRP on each postoperative day.

**Results:**

Thirty-eight patients were included. The majority were male (30, 79%), over 65 years old (30, 79%), with colonic cancer (23, 61%) and node-negative disease (24, 63%). Fourteen patients (37%) had open surgery and 24 (63%) had a laparoscopic resection. A progressive reduction in VO_2_ at peak exercise was significantly associated with both increasing American Society of Anesthesiology (ASA) grade (median, ml/kg/min: ASA 1 = 22, ASA 2 = 19, ASA 3 = 15, ASA 4 = 12, *p* = 0.014) and increasing mGPS (median, ml/kg/min: mGPS 0 = 18, mGPS 1 = 16, mGPS 2 = 14, *p* = 0.039) There was no significant association between either VO_2_ at the AT or peak exercise and postoperative CRP.

**Conclusions:**

The present pilot study reports a possible association between preoperative CPET-derived measures of exercise tolerance, and the preoperative systemic inflammatory response, but not postoperative CRP in patients undergoing surgery for colorectal cancer.

## Background

Colorectal cancer is a leading cause of death in the developed world (Cancer Research UK, 2016). Surgery continues to form the mainstay of treatment in the majority of cases; however, there is a significant associated degree of morbidity and mortality (Ghaferi et al., 2011). Long-term survival is primarily dictated by tumour differentiation and stage at presentation; however, it is increasingly recognised that postoperative complications have a significant impact on long-term oncologic outcomes (McSorley et al., 2016a).

Cardiopulmonary exercise testing (CPET/CPX) has been developed as a method of assessing a patient’s ability to meet the increased oxygen demand of major surgery (Older et al., 1993). It represents a dynamic, non-invasive assessment of a patient’s cardiovascular and pulmonary reserve (Smith et al., 2009). Two key measurements relating to oxygen delivery can be derived via CPET; oxygen consumption at the anaerobic threshold (VO_2_ at AT) which represents the point at which anaerobic metabolism is required in addition to aerobic metabolism to meet tissue energy demand, and oxygen consumption at peak exercise (VO_2_ at peak). Patients with VO_2_ at AT < 11 ml/min/kg or VO_2_ at peak < 19 ml/min/kg are at significant risk of postoperative cardiovascular death and also of surgical complications following major abdominal surgery (Older et al., 1999). Very similar thresholds have also been found to predict the development of postoperative complications in surgery for oesophagogastric cancer (Moyes et al., 2013), in surgery for rectal cancer and colon cancer (West et al., 2014a; West et al., 2014b).

The magnitude of the postoperative systemic inflammatory response is associated with the development of, and severity of, complications following surgery for colorectal cancer (Platt et al., 2012; McSorley et al., 2015). The acute phase reactant C-reactive protein (CRP) has been shown to be a reliable marker of the magnitude of the postoperative systemic inflammatory response (Watt et al., 2015). Indeed, threshold values have been established in the postoperative period which are associated with the development of postoperative complications and the need for investigation (McDermott et al., 2015). The exact mechanism by which poor VO_2_ at AT and VO_2_ at peak are linked to the development of postoperative complications is incompletely understood. It may be that poor cardiopulmonary exercise tolerance leads to the development of postoperative complications due to an exaggerated postoperative systemic inflammatory response.

Therefore, the aim of the present pilot study was to investigate the relationship between CPET measurements, the preoperative systemic inflammatory response as measured by the modified Glasgow Prognostic Score (mGPS), the postoperative systemic inflammatory response as evidenced by CRP, and complications following surgery for colorectal cancer.

## Methods

### Patients

This observational pilot study included patients who had undergone CPET prior to elective surgery for histologically confirmed colorectal cancer in a single centre between September 2008 and April 2017.

All patients received prophylactic antibiotics and venous thromboprophylaxis prior to the induction of anaesthesia as per hospital policy. Further postoperative investigation and intervention was at the discretion of the patient’s surgical team.

### Methods

Clinicopathological data was collected prospectively in a database and anonymised. Recorded information included patient demographics, American Society of Anesthesiology (ASA) grade, body mass index (BMI), smoking status, tumour site, TNM stage (TNM, AJCC), surgical approach, preoperative and postoperative serum CRP and albumin measurements. Data regarding the nature, severity and management of complications was retrospectively categorised using the Clavien Dindo scale (Dindo et al., 2004). Any uncertainties were addressed by review of electronic and/or physical case notes. The study was approved by the West of Scotland Research Ethics Committee, Glasgow.

Serum concentrations of CRP (mg/l) were measured using an autoanalyser (Architect; Abbot Diagnostics, Maidenhead, UK) with a lower detectable limit of 0.2 mg/l as was serum albumin (normal range 35-50 g/l). The preoperative modified Glasgow Prognostic Score (mGPS) was calculated from preoperative serum CRP and albumin (McMillan, 2013).

Cardiopulmonary exercise testing was performed in a single respiratory function laboratory using a ZAN 600 (nSpire Health, Hertford, UK) and Ergoselect bicycle ergometer (Ergoline, Bitz, Germany). A doctor and resuscitation equipment were present during all tests. Several variables were recorded including electrocardiography, blood pressure, oxygen uptake and carbon dioxide output from analysis of inspiratory and expiratory gases. Patients were exposed to an incremental physical exercise protocol to their maximally tolerated level which was determined by exhaustion, symptomatic breathlessness or pain. The measured variables along with the exercise protocol allowed VO_2_ at AT and at peak exercise to be quantified.

### Statistical analysis

In addition to being analysed as continuous variables, patients were grouped according to the previously described thresholds of VO_2_ at AT (< 11 or > 11 ml/min/kg) and at peak exercise (< 19 or > 19 ml/min/kg). Categorical data were compared using the chi-square test or Fisher’s exact test where appropriate. Continuous data are presented as median and range and were compared using the Mann-Whitney *U* test or Kruskal-Wallis test in multiple groups. Postoperative CRP concentrations were displayed graphically by postoperative day as median and 95% confidence interval. Correlation between VO_2_ at AT and VO_2_ at peak exercise and the peak postoperative CRP concentration was assessed using Spearman’s correlation coefficients. Statistical analyses were performed using IBM SPSS version 22 for Windows (Chicago, IL, USA).

## Results

### Patients

Thirty-eight patients completed CPET prior to elective surgery for colorectal cancer at Glasgow Royal Infirmary between 2008 and 2017 (Table 1). The majority were male (30, 79%), over 65 years old (30, 79%), with colonic cancer (23, 61%) and node-negative disease (24, 63%). Fourteen patients (37%) had open surgery and 24 (63%) had a laparoscopic resection. Prior to surgery, 3 patients with locally advanced or margin threatening rectal cancer underwent neoadjuvant chemoradiotherapy (nCRT); there were no cases of pathological complete response.

**Table 1:** Patient characteristics and postoperative C-reactive protein levels grouped by VO_2_ at the anaerobic threshold and at peak exercise

Characteristic	Cardiopulmonary exercise test variable					
	VO_2_ at AT < 11 ml/kg/min (n)	VO_2_ at AT > 11 ml/kg/min (n)	*P*	VO_2_ at peak < 19 ml/kg/min (n)	VO_2_ at peak > 19 ml/kg/min (n)	*P*
Preoperative						
Age (< 65/65–74/> 74)	5/8/11	3/7/4	0.488	4/9/12	4/6/3	0.130
Sex (male/female)	19/5	11/3	1.000	19/6	11/2	0.689
ASA (1/2/3/4)	0/11/12/1	2/8/4/0	0.041	0/10/14/1	2/9/2/0	0.004
BMI (< 20/20–25/36–30/> 30, kg/m^2^)	1/4/7/12	1/4/5/4	0.206	1/4/7/13	1/4/5/3	0.106
Smoker (never/ex/current)	11/9/4	6/6/2	0.981	10/12/3	10/12/3	0.912
Site (colon/rectum)	16/8	7/7	0.492	17/8	6/7	0.295
TNM stage (I/II/III/IV)	2/12/9/0	3/7/3/1	0.510	3/13/9/0	2/6/3/1	0.969
Preop mGPS (0/1–2)	14/8	11/2	0.259	13/9	12/1	0.036
Neoadjuvant (yes/no)	1/23	2/12	0.542	0/25	3/10	0.034
Intraoperative						
Approach (open/lap)	6/18	8/6	0.081	4/21	10/3	< 0.001
Stoma (yes/no)	10/13	6/8	1.000	11/13	5/8	0.666
Transfusion (yes/no)	1/20	0.10	1.000	1/20	0/10	1.000
Surgery > 4 h (yes/no)	14/10	6/8	0.503	16/9	4/9	0.087
Postoperative						
Any complication (yes/no)	9/15	6/8	1.000	10/15	5/8	1.000
Clavien Dindo grade 3–5(yes/no)	8/16	2/12	0.268	2/23	1/12	1.000
Length of stay (median, range, days)	8 (3–19)	8 (5–15)	0.790	8 (3–15)	9 (5–19)	0.169
POD 3 CRP > 150 mg/l (yes/no)	11/12	8/4	0.476	12/12	7/4	0.493
POD 4 CRP > 150 mg/l (yes/no)	5/12	5/9	0.709	6/13	4/8	0.919

*ASA* American Society of Anesthesiology, *BMI* body mass index, *AT* anaerobic threshold, *mGPS* modified Glasgow Prognostic Score, *POD* postoperative day, *CRP* C-reactive protein

### Complications

Of the 38 patients, 15 (39%) experienced complications (Table 1). No patients died within 30 days of surgery or during the same admission. Of the patients with complications, 10 (26% of all patients) were infective and 5 (13%) were non-infective. When classified using the Clavien Dindo scale, 12 (32% of all patients) were grade 1 to 2 (i.e. required minor intervention) and 3 (8%) were grade 3 to 4 (i.e. necessitated major intervention).

### Associations between CPET variables, co-morbidity and mGPS

**Fig. 1:**
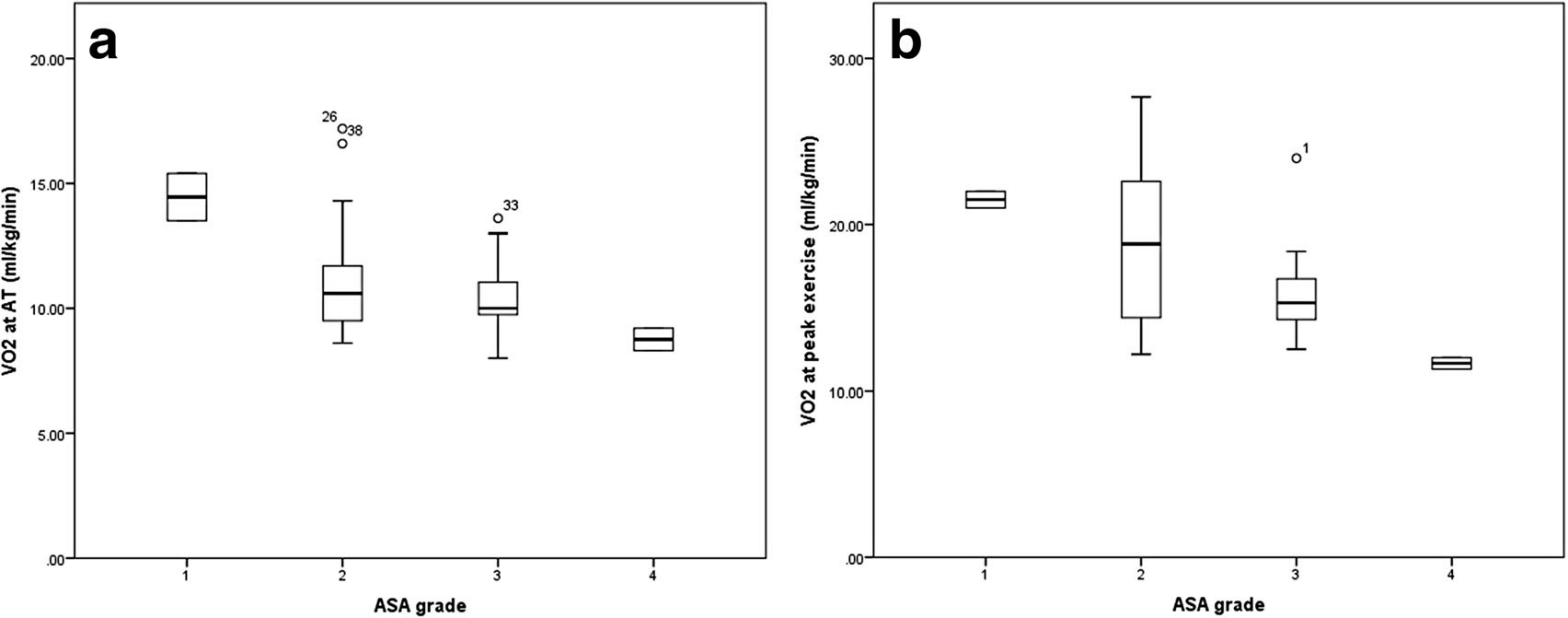
Boxplots of **a** VO_2_ at anaerobic threshold (ml/kg/min) and **b** VO_2_ at peak exercise (ml/kg/min) grouped by American Society of Anesthesiology (ASA) grade

There was a significant positive correlation (r_s_ = 0.628, *p* < 0.001) between VO_2_ at anaerobic threshold (AT) and VO_2_ at peak exercise. An increasing burden of co-morbidity as measured by ASA grade (Fig. 1) was significantly associated with progressively lower median VO_2_ at peak exercise (ml/kg/min: ASA 1 = 22, ASA 2 = 19, ASA 3 = 15, ASA 4 = 12, *p* = 0.014), but not VO2 at AT (*p* = 0.058).

**Fig. 2:**
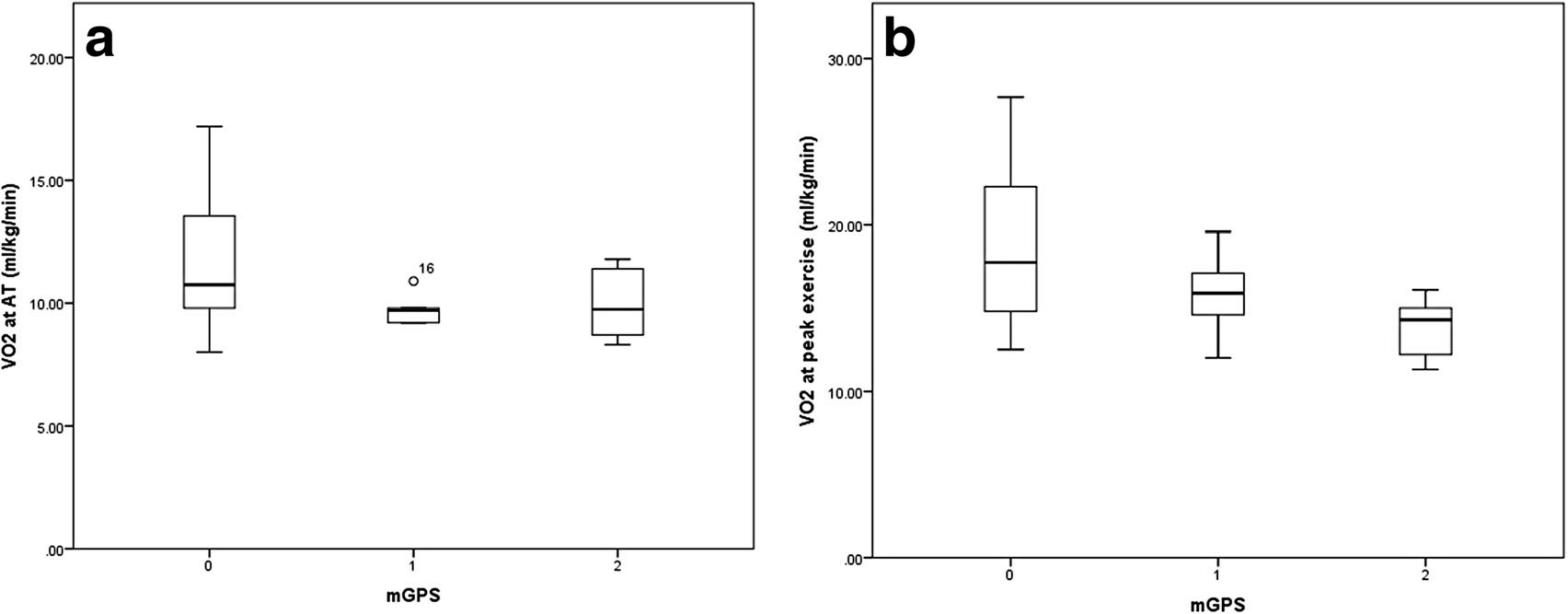
Boxplots of **a** VO_2_ at anaerobic threshold (ml/kg/min) and **b** VO_2_ at peak exercise (ml/kg/min) grouped by modified Glasgow Prognostic Score (mGPS)

When VO_2_ at AT was compared as a continuous variable amongst patients grouped by preoperative mGPS 0, 1 and 2 (Fig. 2), there was no significant association (*p* = 0.147). When VO_2_ at peak exercise was compared as a continuous variable amongst patients groups by mGPS 0, 1 and 2 (Fig. 2), higher mGPS was significantly associated with progressively lower median VO_2_ at peak exercise (ml/kg/min: mGPS 0 = 18, mGPS 1 = 16, mGPS 2 = 14, *p* = 0.039).

There was a non-significant linear trend toward greater preoperative systemic inflammation in patients with higher ASA grade (*p* = 0.058).

### VO_2_ at anaerobic threshold and the postoperative SIR

Fourteen patients (37%) had VO_2_ at AT > 11 ml/min/kg and 24 patients (63%) had VO_2_ at AT < 11 ml/min/kg (Table 1). When the two groups were compared, there was a significant association between VO_2_ at AT and ASA grade (*p* = 0.041). There was no significant association between VO_2_ at AT and other preoperative characteristics including patient age, sex, BMI, smoking status, tumour site, TNM stage, preoperative mGPS or neoadjuvant treatment (Table 1).

**Fig. 3:**
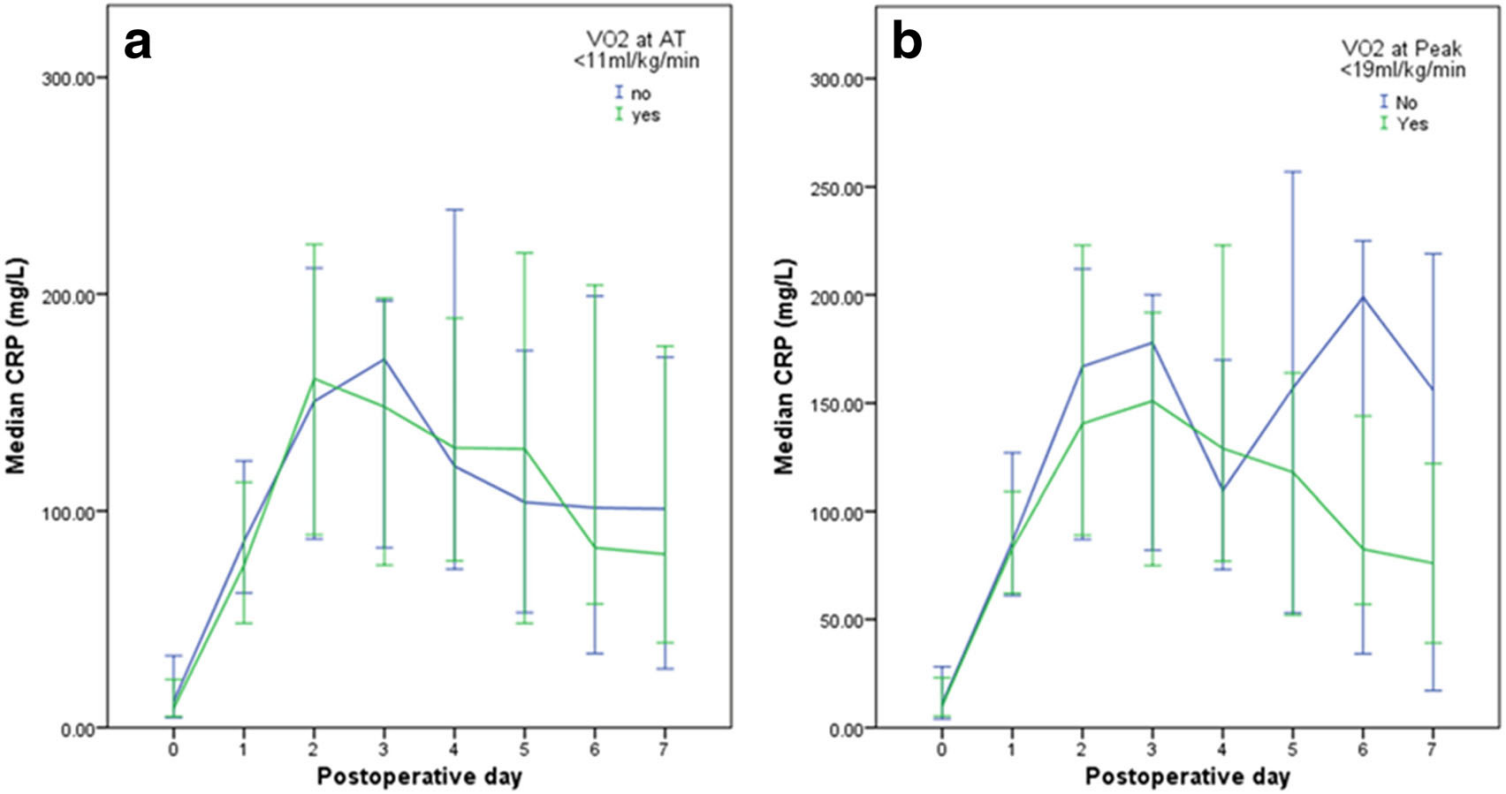
Median postoperative C-reactive protein (CRP) concentrations (mg/l) in patients grouped by **a** VO_2_ at the anaerobic threshold (ml/kg/min) and **b** VO_2_ at peak exercise (ml/kg/min)

There were no significant associations between VO_2_ at AT and postoperative complications, established CRP thresholds on postoperative days 3 or 4 (Table 1), or the postoperative CRP trend (Fig. 3). When both VO_2_ at AT and peak postoperative CRP (day 4) concentrations were compared as continuous variables, there was no significant correlation (*p* = 0.885).

### VO_2_ at peak exercise and the postoperative SIR

Thirteen patients (34%) had VO_2_ at peak exercise > 19 ml/min/kg and 25 patients (66%) had VO_2_ at peak exercise < 19 ml/min/kg (Table 1). When the two groups were compared (Table 1), there was a significant association between VO_2_ at peak exercise and ASA (*p* = 0.004). A significantly higher proportion of patients with VO_2_ at peak exercise < 19 ml/min/kg had an mGPS of 1–2 (41 vs. 8%, *p* = 0.036). A significantly lower proportion of patients with VO_2_ at peak exercise < 19 ml/min/kg underwent nCRT (0 vs. 23%, *p* = 0.034). With regard to intraoperative variables (Table 1), a significantly higher proportion of patients with VO_2_ at peak exercise < 19 ml/min/kg underwent laparoscopic surgery (84 vs. 23%, *p* < 0.001).

There was no significant association between VO_2_ at peak exercise and postoperative complications, established CRP thresholds on postoperative days 3 or 4 (Table 1), or the postoperative CRP trend (Fig. 3). When VO_2_ at peak exercise and peak postoperative CRP (day 3) concentrations were compared as continuous variables, there was no significant correlation (*p* = 0.898).

## Discussion

The present pilot study confirms the relationship between CPET-derived measures of exercise tolerance and co-morbidity as measures by ASA grade in patients prior to surgery for colorectal cancer. Moreover, the present results show for the first time an inverse relationship between the VO_2_ at peak exercise and the preoperative systemic inflammatory response. There was no significant association with the magnitude of the postoperative systemic inflammatory response. However, given the small numbers of patients examined, these relationships warrant further investigation.

The neuroendocrine, metabolic and immune responses to surgical trauma lead to an increase in oxygen requirement from baseline usually supplied by increasing tissue oxygen extraction and cardiac output in the postoperative period, with the aim of increasing oxygen delivery (Shoemaker & Czer, 1979). However, not all patients are able to utilise these mechanisms sufficiently to prevent the accrual of an “oxygen debt”, when oxygen delivery is outstripped by tissue oxygen requirement (Waxman et al., 1981). The degree of oxidative stress placed on the patient has been found to be associated with the production of pro-inflammatory cytokines (Rixen & Siegel, 2000). It has been postulated that oxidative stress and resultant tissue hypoxia, especially in the gut, drives a significant proportion of the postoperative systemic inflammatory response (Mainous et al., 1995). Indeed, it is well recognised that tissue hypoxia can lead to activation and augmentation of the innate immune system via hypoxia-inducible factor 1α (HIF-1α) (Peyssonnaux et al., 2005; Nizet & Johnson, 2009). CPET thus uses graded exercise to quantify a given patients’ anaerobic threshold and other measures including VO_2_ at peak exercise and METs. These CPET variables are associated with postoperative outcomes following abdominal and colorectal surgery (Older et al., 1999; West et al., 2014a; West et al., 2014b).

It was of interest that a significant association was found between VO_2_ at peak exercise and the preoperative mGPS at the univariate level. It remains unclear whether this relationship is explained by the association between preoperative systemic inflammation and co-morbid state or other effects. Indeed, the preoperative systemic inflammatory response has previously been shown to be directly associated with preoperative co-morbidity in patients undergoing surgery for colorectal cancer (Richards et al., 2010), and it may be this which links mGPS to reduced peak exercise tolerance. This finding was not confirmed by the results of the present study. However, the trend to association between mGPS and ASA was likely non-significant due to patient numbers. Alternatively, systemic inflammation has a key causal role in the development of the cancer cachexia syndrome, with loss of skeletal muscle quantity and quality, and resultant loss of physical function in patients with cancer (McSorley et al., 2017a). It may be that systemic inflammation exerts its influence on exercise tolerance through this mechanism.

Although previous studies in colorectal surgery have reported an association between patients with VO_2_ at AT < 11 ml/min/kg and VO_2_ at peak exercise < 19 ml/min/kg and the development of postoperative complications (West et al., 2014a; West et al., 2014b), this was not confirmed in the present study. This is most likely due to the small number of patients in the present study. The magnitude of the postoperative systemic inflammatory response, as evidenced by CRP, is increasingly understood to be associated with the development of postoperative complications following surgery for colorectal cancer (Watt et al., 2017a). These postoperative complications, whether categorised by their type or severity, are associated with poorer long-term oncologic outcomes following surgery for colorectal cancer (McSorley et al., 2016a). Furthermore, studies in surgery for oesophageal, gastric and colorectal cancer suggest that the magnitude of the postoperative systemic inflammatory response is itself a prognostic factor (Matsuda et al., 2015; Saito et al., 2015; McSorley et al., 2016b). Such findings have prompted the investigation of patient and operative factors which influence the postoperative systemic inflammatory response, along with potential methods which might be used to attenuate it, with the aim of reducing postoperative complication rates (Watt et al., 2017b; McSorley et al., 2016c; McSorley et al., 2017b). Indeed, it might be hoped that ongoing studies examining prehabilitation as an intervention to reduce postoperative complication rates may also find an impact on the postoperative systemic inflammatory response.

The main limitation of the present study is the small number of included patients. Preoperative CPET is not routinely used as an evaluation of fitness for colorectal surgery in our unit at present. These small numbers lead to limited ability to make confident statements about the association between CPET, postoperative CRP and complications, and prevented subgroup analysis. Furthermore, the multiple comparisons used in analysis, in the context of the small number of patients, also reduce the confidence in the observed associations or lack thereof.

## Conclusions

In conclusion, the present pilot study reports a possible association between preoperative CPET-derived measures of exercise tolerance, and the preoperative systemic inflammatory response in patients undergoing surgery for colorectal cancer. The mGPS may be a surrogate for overall “fitness” in these patients; however, systemic inflammation may well be a causal factor in poor exercise tolerance in this group of patients. No association was found between CPET-derived measures and the magnitude of the postoperative systemic inflammatory response; however, small numbers and the presence of important confounders mean that further work in a larger cohort of patients is warranted.

## Abbreviations

AJCC: American Joint Committee on Cancer
ASA: American Society of Anesthesiology
AT: Anaerobic threshold
BMI: Body mass index
CPET/CPX/CPEX: Cardiopulmonary exercise testing
CRP: C-reactive protein
HIF: Hypoxia inducible factor
MET: Metabolic equivalent
mGPS: Modified Glasgow Prognostic Score
nCRT: Neoadjuvant chemoradiotherapy
TNM: Tumour nodes metastasis staging
VO_2_: Volume of oxygen consumed
